# Clinical and Molecular Findings in *PROM1*-Associated Inherited Retinal Dystrophies

**DOI:** 10.3390/genes16111299

**Published:** 2025-11-01

**Authors:** Fabiana D’Esposito, Caterina Gagliano, Sabrina Vallone, Francesco Cappellani, Giuseppe Gagliano, Viviana Randazzo, Daniele Tognetto, Gabriella Esposito, Marco Zeppieri

**Affiliations:** 1 Imperial College Ophthalmic Research Group (ICORG) Unit, Imperial College, 153-173 Marylebone Rd, London NW15QH, UK; f.desposito@imperial.ac.uk; 2 Department of Medicine and Surgery, University of Enna “Kore”, Piazza dell’Università, 94100 Enna, Italy; 3 Eye Center “G.B. Morgagni-DSV”, 95125 Catania, Italy; 4 Integrated Care Department of Laboratory and Transfusion Medicine, University Hospital Federico II, 80131 Naples, Italy; 5 Eye Clinic, Catania University, Viale Carlo Azeglio Ciampi, 95121 Catania, Italy; 6 Ophthalmic Unit, A.O.R. Villa Sofia-Cervello, 90100 Palermo, Italy; 7 Department of Medicine, Surgery and Health Sciences, University of Trieste, 34127 Trieste, Italy; 8 Department of Molecular Medicine and Medical Biotechnologies, University of Naples Federico II, 80100 Naples, Italy; 9 CEINGE-Advanced Biotechnologies Franco Salvatore, 80100 Naples, Italy; 10 Department of Ophthalmology, University Hospital of Udine, 33100 Udine, Italy

**Keywords:** genotype-phenotype correlation, inherited retinal dystrophies, genetic counseling, retinal degeneration

## Abstract

**Background:** Inherited retinal dystrophies (IRDs) include a clinically and genetically diverse array of conditions resulting in progressive visual impairment. The *PROM1* gene is crucial for the development and maintenance of photoreceptors. Variants in *PROM1* are linked to a wide phenotypic spectra of IRDs; however, the correlation between genotype and phenotype is not fully elucidated. Comprehending these relationships is essential for enhanced diagnostic precision, patient guidance, and formulation of focused treatments. **Objective:** This study aims to examine the genotype–phenotype associations in patients with *PROM1*-associated IRDs. Clinical variability and inheritance patterns linked to different pathogenic variants are examined, aiming to clarify their different behaviors. **Methods:** We performed a retrospective investigation of patients identified as affected by *PROM1*-related IRDs. Thorough ophthalmologic assessments, including retinography, fundus autofluorescence, optical coherence tomography (OCT), and electrodiagnostic testing (EDT), were conducted. Genetic testing was performed via targeted gene panels or whole-exome sequencing. Variants were categorized based on ACMG criteria, and inheritance patterns were determined by familial segregation analysis. Clinical characteristics were analyzed among genotypic groups to ascertain potential phenotype–genotype relationships. **Results:** All patients had pathogenic or likely pathogenic *PROM1* mutations. Both autosomal dominant and autosomal recessive inheritance patterns were identified. Dominant pathogenic variants were predominantly linked to late-onset cone-rod dystrophy or macular dystrophy, whereas biallelic variants frequently resulted in early-onset severe rod–cone dystrophy characterized by fast vision deterioration. A group of patients with the same genotypes displayed significant phenotypic variability, indicating the potential impact of modifier genes or environmental influences. Truncating mutations in the N-terminal region were significantly associated with earlier illness onset and greater functional impairment. **Conclusions:** *PROM1*-related IRDs demonstrated significant clinical and genetic heterogeneity, with the route of inheritance and type of variant affecting disease severity and progression. Our findings underscore the significance of thorough genotypic and phenotypic characterization in afflicted individuals. A deeper comprehension of *PROM1*-related IRD disease pathways can enhance prognosis, direct clinical care, and facilitate the advancement of genotype-based therapy strategies.

## 1. Introduction

Inherited retinal dystrophies (IRDs) encompass a wide spectrum of genetically determined conditions, leading to usually severe visual impairment. They are characterized by a pronounced high genetic and phenotypic heterogeneity [1,2,3]. Several classifications have been proposed based on the initial and prevalent photoreceptor system involved (cones and/or rods), the type of transmission, or the retinal areas involved (macula and/or periphery) [4]. At present about 300 genes have been characterized as underlying different types of IRDs, and a consistent number of those display a high phenotypic heterogeneity, potentially being related to isolated or syndromic forms, or to retinal diseases with very different characteristics [3].

One of the genes displaying the highest heterogeneity in the related phenotypes and in the mode of transmission is *PROM1* (OMIM * 604365) [3,5]. *PROM1* encodes prominin-1, a pentaspan transmembrane glycoprotein with a crucial role in the formation and organization of disks within the outer segment of photoreceptors [6,7]. Further studies have demonstrated the role of PROM1 in regulating autophagosome maturation and trafficking within retinal pigment epithelium (RPE) cells [8]. Recent studies on animal models have confirmed its role in RPE homeostasis and retinal health, providing insights on considering *PROM1* a target for potential future therapeutic strategies, not only for IRDs, but also for age-related macular degeneration (AMD) [9].

The prevalence of *PROM1*-related IRDs varies according to different studies in different populations, but it can be considered relatively rare, being evaluated to be the underlying cause of approximately 1–2% of inherited retinal dystrophies [10,11]. *PROM1*-related IRDs display different patterns of inheritance, both dominant and recessive [12]. The pathogenetic mechanisms of the different variants of *PROM1* acting in a dominant or recessive manner are still poorly understood, but alternative splicing defects of mutated *PROM1* seem to play an important role [13]. *PROM1* pathogenic variants are related to a variety of retinal phenotypes, including autosomal recessive rod–cone dystrophy (arRCD) or retinitis pigmentosa (RP) [14,15] with macular atrophy [16]; autosomal dominant cone–rod dystrophy (adCRD) with macular dystrophy (MD) [6], such as the Stargardt-like form, also defined as STG4 phenotype [17]; and maculopathies displaying a “bull’s eye” aspect [12,18,19].

The nucleotide change c.1117C>T (p.Arg373Cys) is the most prevalent dominantly transmitted variant across different populations, but the proportion of patients seems higher in Asian, compared to Western, populations [20,21,22]. The progression of retinal dysfunction and degeneration varies greatly according to the mode of inheritance and the type of pathogenic variants. Generally, dominantly transmitted variants display a slower progression of visual loss compared to the recessive forms [23,24].

In our study, we describe a series of patients affected by *PROM1*-related IRDs in an attempt to establish a genotype–phenotype correlation, with the aim of enhancing understanding of the natural history and pathogenic effects of this heterogeneous disease-related gene.

## 2. Patients and Methods

The analysis is based on a retrospective study of 10 Italian patients affected by retinal dystrophies who underwent a comprehensive ophthalmologic examination and had a confirmed molecular diagnosis of *PROM1*-related IRD. This was a retrospective observational study that consisted of collecting data from non-invasive diagnostic examinations, which are commonly performed on most patients. Patients underwent routine and standardized non-invasive diagnostic testing. Special IRB approval was not required and was waived by the Institutional Review Board and the ethics committee. This study was performed according to the ethical standards of our Institutional Review Board and in accordance with the ethical standards laid down in the 1964 Declaration of Helsinki and its later amendments.

Patients were diagnosed at different clinical institutions with various equipment. Their comprehensive ophthalmic examination included best-corrected visual acuity (BCVA), intraocular pressure (IOP) measurement (noncontact tonometer), slit-lamp anterior and posterior segment biomicroscopy, color fundus photography, autofluorescence, OCT, and dark- and electrodiagnostic testing (EDT). When not performed personally by any of the authors of this study, provided documentation was evaluated to establish or confirm a diagnosis. Pedigrees were defined through interviews with patients and their family members. When doubts arose about the affected/unaffected status of a family member, examination was provided.

Patients were analyzed in different centers, all with NGS technology, focusing on IRDs-related genes. Identified potentially pathogenic variants were confirmed through Sanger sequencing, and familial segregation confirmed the genetic diagnosis. Affected family members were analyzed through NGS to investigate the presence of possible additional variants with a modifier effect. The pathogenic role of the identified variants was predicted using the bioinformatic prediction tool Varsome (https://varsome.com/, accessed on 1 September 2025), which classifies variants according to the American College of Medical Genetics and Genomics standards and guidelines [25,26].

## 3. Results

Table 1 lists the sequence variants identified in the *PROM1* gene of the patients (nine males and one female) described in this study.

Demographic characteristics, symptoms, clinical findings, and genotypes of participants are summarized in Table 2.

Only two patients in our cohort were related (son and father, patients #2 and #3, respectively), but displayed different phenotypes in relation to the different genotypes, and have already been described in a previous publication by our group [27]. Six patients (# 2, 3, 6, 7, 9, and 10) carried the dominant variant c.1117C>T, but patient # 2 displayed a far more severe phenotype derived from the presence of an additional variant (c.1142-1G>A) inherited from the unaffected mother and therefore considered recessive. Patient # 10 also carried two variants of uncertain significance (VUS) in the *CDH23* gene (c.6614C>T and c.9569C>T). Four patients (# 1, 4, 5, and 8) displayed recessive inheritance patterns. In particular, one patient was homozygous for the c.1414C>T variant, and the other had compound heterozygous genotypes, namely c.1354dupT/c.1405C>A, c.1354dup/c.1414C>T, and c.436C>T/c.1321_1330dup.

## 4. Discussion

The phenotypes of our patients varied greatly. Generally, patients affected by the c.1117C>T-related dominant form displayed less severe phenotypes, mainly characterized by late-onset MD and nyctalopia. A more severe phenotype was associated with the presence of an additional pathogenic variant in compound heterozygosis in patient #2, a male aged 22, manifesting visual impairment since pre-school age. At observation, his visual acuity was hand motion (HM) in both eyes. In his clinical history, he reported nyctalopia in the first phases, together with photophobia and progressive visual decay. His father, aged 51, displayed far less severe MD compared to his son, with visual impairment beginning in his 3rd decade, and at our observation being 6/9 in OU. While both patients were carrying the c.1117C>T variant, the son also carried a c.1142–1G>A maternally inherited variant, recessively acting in the mother, who was completely unaffected at examination. Our hypothesis is that the additional variant in patient # 2 can be responsible for his more severe phenotype compared to his father [27].

Interestingly, patient # 5 was compound heterozygous for the known recessive pathogenic variant c.1354dupT and the novel missense variant c.1405C>A, the only VUS identified in our patients. The c.1405C>A variation has not previously been documented in people with PROM1-related inherited retinal diseases (IRDs). The bioinformatic assessment by VarSome (accessed on 1 October 2025) initially categorized it as a Variant of Uncertain Significance (VUS). In accordance with ACMG/AMP standards [26], we have methodically implemented the subsequent criteria: PM2 (moderate)—exceedingly low frequency in gnomAD (MAF 0.0000144), PP2 (supporting)—missense variant in a gene exhibiting low tolerance for benign variation, PP3 (supporting)—numerous in silico methods forecasting a harmful effect, PM3 (moderate)—identified in trans with a recognized pathogenic allele (c.1354dupT), and PP1 (supportive)—cosegregation with the disease within the family. The utilization of two moderate criteria (PM2 and PM3) and three supporting criteria (PP1, PP2, and PP3) satisfies ACMG/AMP guidelines for categorization as ‘likely pathogenic.’ Gene-specific factors for PROM1, based on previous studies [12], reinforce this interpretation by highlighting the pathogenic significance of variants impacting conserved extracellular domains of PROM1. We have thus determined that c.1405C>A (p.Pro469Thr) constitutes a novel, likely pathogenic allele with a comparatively mild impact on protein function, aligning with the delayed disease onset noted in our patient. Nevertheless, we considered the c.1405C>A variant with a relatively mild effect on protein function, as suggested by the late onset of the disease in the patient, compared to the other patients with *PROM1*-related recessive IRDs (Table 2).

**Figure 1:**
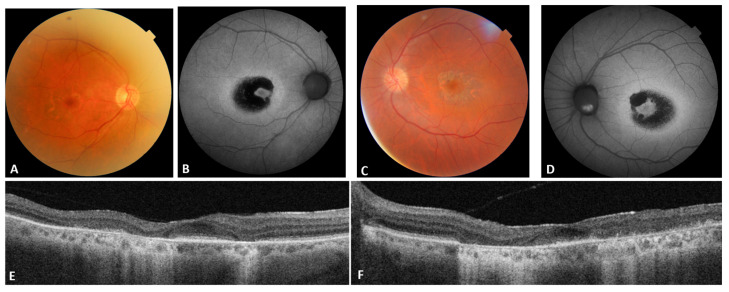
Patient #7 multimodal imaging. (**A**,**C**): OD and OS color fundus; (**B**,**D**): OD and OS blue autofluorescence; (**E**,**F**): OD and OS OCT.

Patient #7 displayed a later onset of symptoms and, at multimodal imaging, a small island of preserved retinal layers in the foveal area (Figure 1).

Patient #10 had a more severe phenotype than expected, also compared to his affected family members. Two variants of uncertain significance (VUS) in *CDH23* were found in a heterozygous form lacking phasing or segregation evidence. The recessive nature of *CDH23*, a gene related to Usher Syndrome [3], along with the lack of audiometric anomalies, rendered the existing evidence inadequate to substantiate a modifier impact. Consequently, we classified these variations as accidental observations with ambiguous clinical relevance.

In dominant cases, OCT generally demonstrated localized disruption of the ellipsoid zone and fundus autofluorescence exhibited parafoveal rings of hyperautofluorescence surrounding the afflicted macula. In recessive cases, OCT exhibited more extensive and confluent outer retinal atrophy, frequently accompanied by early foveal thinning, while autofluorescence displayed significant hypoautofluorescence indicative of widespread retinal pigment epithelium malfunction.

Patients #2 and #10 displayed unexpectedly severe symptoms. In patient #2 the explanation is likely to reside in the presence of an additional pathogenic variant that, although behaving in a recessive manner, can aggravate the phenotype of a dominant heterozygous variant. In the case of patient #10, the characteristics of the gene where the additional variants are found lead us to exclude its role in the severity of the phenotype, suggesting the potential involvement of additional genetic or epigenetic modifiers affecting illness manifestation. The significance of modifier genes in hereditary retinal dystrophies is gaining acknowledgment, with new evidence indicating that allelic interactions, gene–gene communication, and polygenic risk may influence phenotypic variability. Although we cannot reach conclusive determinations in our sample, these data highlight the importance of accounting for modifier effects in the interpretation of clinical outcomes.

Genotype–phenotype diversity in *PROM1*-related dystrophies has been observed in several populations. The c.1117C>T variant appears to be prevalent in most populations, usually giving rise to MD associated with nyctalopia [24]. Transmission of *PROM1* pathogenic variants is well described as possibly being both autosomal dominant and recessive, and phenotypes are knowingly extremely variable. Our findings, especially the discovery of new recessive variations, underscore extensive clinical heterogeneity and indicate that *PROM1*-related IRDs cannot be well represented by a singular diagnostic definition. From a potentially therapeutic point of view, our findings underscore the significance of early genetic testing for patients exhibiting unusual or ambiguous presentations, as prompt molecular diagnosis might enhance patient counseling and enable stratification for clinical trials aimed at specific molecular subgroups. Furthermore, the role of additional variants can have a clinically relevant effect, with next-generation sequencing (NGS) being the most indicated diagnostic tool, even in the presence of a known familial pathogenic variant that could normally be investigated by direct sequencing. Finally, the identification of new variations broadens the mutational spectrum of *PROM1* and contributes to the evidence required for forthcoming functional and therapeutic investigations.

The novel recessive variants presented here impact residues that are highly conserved among vertebrates, indicating functional significance. In silico structural predictions suggest possible interference with extracellular loop domains critical for *PROM1*’s function in photoreceptor disk formation. These characteristics enhance the pathogenic potential of the variations and underscore the importance of their detection.

In our cohort, individuals carrying dominantly inherited *PROM1* pathogenic variants typically exhibited a later development of macular degeneration and nyctalopia compared to those with recessive patterns. These observations, however indicative, should be interpreted cautiously due to the limited sample size and lack of statistical analysis. To prevent overgeneralization, we have articulated these findings as descriptive patterns rather than conclusive associations.

This study highlights the practical implications of genotype–phenotype diversity for therapeutic therapy. The clinician’s capacity to predict illness progression and customize recommendations is frequently constrained due to patients presenting with common characteristics. Nonetheless, a systematic methodology that incorporates longitudinal follow-up, visual function evaluation, and family history analysis can still offer significant direction in routine practice. The acknowledgment that people with identical pathogenic variants may display varying disease manifestations underscores the necessity of tailored patient care over a uniform approach. Furthermore, these findings necessitate enhanced communication tactics with patients and families, ensuring their awareness of both the possibility for disease progression and the prospects for future involvement in clinical research. This amalgamation of clinical vigilance and patient-centered discourse can ultimately enhance quality of life, especially in the absence of immediate therapeutic alternatives.

The limited cohort size has been the primary constraint of this investigation, hindering the generalizability of the findings and preventing rigorous statistical analyses. A multicenter collaboration would have been the ideal strategy for obtaining more significant and clinically important results. The current study provides preliminary insights into an Italian population and emphasizes the necessity of extensive future investigations that can include and assess data across different centers.

This study possesses many intrinsic limitations that require acknowledgment. The limited sample size constrains the generalizability of the results and hinders the establishment of statistically meaningful correlations between particular variations and clinical traits. Furthermore, as this constitutes a retrospective investigation, data consistency is partially affected by variations in equipment and testing techniques among the participating institutions. A further disadvantage is the lack of long-term longitudinal data for all patients, which would be useful in elucidating the natural history of *PROM1*-related dystrophies. Notwithstanding these limitations, this study offers significant insights that may inform future research endeavors. Future multicenter partnerships involving larger cohorts, consistent imaging and functional evaluations, and the incorporation of new molecular methodologies are essential to enhance genotype–phenotype correlations. Moreover, the investigation of modifier genes, environmental factors, and novel therapeutic strategies, such as gene- and cell-based therapies, signifies intriguing avenues for enhancing patient outcomes.

## 5. Conclusions

In our cohort of patients affected by *PROM1*-related IRDs, the pathogenic c.1117C>T variant accounts for 30% of the analyzed alleles, thereby resulting in the most common dominant disease-causing allele. Moreover, we identified two novel recessive pathogenic null variants, namely c.1321_1330dup and c.1414C>T, which are associated with the most severe phenotypes observed in our patients. Lastly, the rare novel disease-causing missense variant c.1405C>A can be considered a hypomorphic allele that, as already shown for other autosomal recessive inherited diseases [28], by combining with a loss-of-function recessive allele in trans, makes the disorder penetrant, with a relatively mild, rather than absent, phenotype.

## Figures and Tables

**Table 1:** Identified variants in the cohort of *PROM1*-related IRD patients.

Nucleotide Variant	Protein Effect	dbSNP Ref. no.	MAF	ACGM	
GenBank no. NM_006017.3				Pathogenicity Criteria	Classification
c.436C>T	p.Arg146*	rs780697796	0.00003	PS4, PVS1, PM2	Pathogenic
c.1117C>T	p.Arg373Cys	rs137853006	0.0000014	PS4, PM2	Likely pathogenic
c.1142-1G>A	splicing	rs752619497	0.0000486	PS4, PVS1, PM2	Pathogenic
**c.1321_1330dup**	p.Leu444Hisfs*24	NR	--	PVS1, PM2	Likely pathogenic
c.1354dupT	p.Tyr452Leufs*13	rs543698823	0.000222	PS4, PVS1, PM2	Pathogenic
**c.1405C>A**	p.Pro469Thr	rs751216717	0.0000144	PM2, PM3, PP1, PP2, PP3 [S1]	Likely pathogenic
**c.1414C>T**	p.Arg472*	rs761152494	0.0000180	PS4, PVS1, PM2	Pathogenic

dbSNP, https://www.ncbi.nlm.nih.gov/snp (accessed 1 October 2025); MAF, dbSNP minor allele frequency; ACGM, American College of Medical Genetics and Genomics; NR, not reported. In bold, variants not previously reported in individuals affected with *PROM1*-related conditions. S1, Supplementary Figure S1.

**Table 2:** Patients’ demographics, phenotype, and genotype.

Patient no.	Sex	Age at Observation	Age at Onset	Duration of Symptoms	BCVA	Symptoms/ Phenotype at Observation	Genotype
1	M	45	25	20	6/60 OU	RCD + MDY	c.1414C>T (homozygous)
2	M	22	4	18	HM OU	RCD + MDY	c.1142-1G>A c.1117C>T
3	M	51	32	19	6/9 OU	MDY	c.1117C>T (heterozygous)
4	M	16	4	12	6/30 OD 6/15 OS	RCD Myopia (−8 D OU) Nyctalopia	c.436C>T c.1321_1330dup
5	F	52	49	3	6/9 OU	MDY Nyctalopia	c.1354dupT c.1405C>A
6	M	53	38	15	HM OD 6/60 OS	MDY	c.1117C>T (heterozygous)
7	M	60	50	10	6/24 OD 6/30 OS	MDY Nyctalopia	c.1117C>T (heterozygous)
8	M	56	12	44	HM OU	RCD MDY	c.436C>T c.1321_1330dup
9	M	40	25	15	LP OD 6/9 OS	MDY Nyctalopia Myopia (−12 D OU) RD OD	c.1117C>T (heterozygous)
10	M	20	14	6	6/9 OD 6/18 OS	MDY Amblyopia OS	c.1117C>T (heterozygous)

M: male. F: female. OD: right eye. OS: left eye. OU: both eyes. RCD: rod–cone dystrophy. MDY: macular dystrophy. D: diopters. RD: retinal detachment.

## Data Availability

The original contributions presented in this study are included in the article/Supplementary Material. Further inquiries can be directed to the corresponding author.

## Supplementary Materials

The following supporting information can be downloaded at https://www.mdpi.com/article/10.3390/genes16111299/s1. Figure S1: Screenshot of the Varsome (accessed on 1 October 2025) prediction obtained for the c.1405C>A variant. Also considering the PM3 and PP1 criteria, it is classified as likely pathogenic.
