# Supplementary material for: Clinical and Molecular Findings in PROM1-Associated Inherited Retinal Dystrophies
Source: Genes (Basel). 2025 Nov 1;16(11):1299. doi: 10.3390/genes16111299 (PMC12652650; doi:10.3390/genes16111299)
Supplement: Supplementary file 1 [file genes-16-01299-s001.zip › genes-3904294-supplementary.pdf]

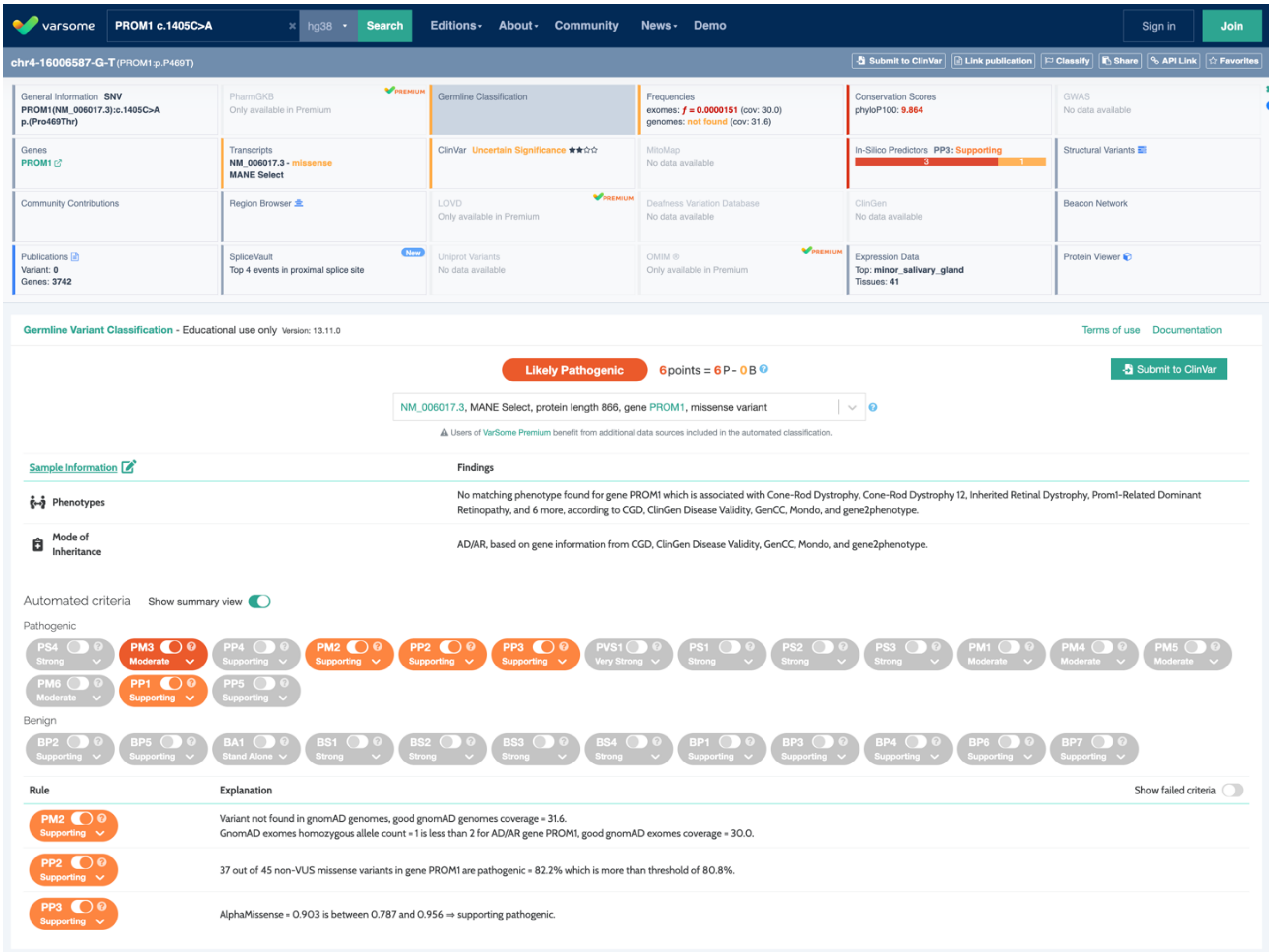

Supplementary Figure S1. Screenshot of the Varsome (accessed on 1 October 2025) prediction obtained for the c.1405C>A variant. Considering also the PM3 and PP1 criteria, it is classified as likely pathogenic.
